# Respiratory Symptoms Correlating to Smoking Prevalence: The National Nutrition Survey and the National Life-style Survey in Japan.

**DOI:** 10.2188/jea.13.226

**Published:** 2007-11-30

**Authors:** Shinichi Asahi, Ritei Uehara, Makoto Watanabe, Morihiro Tajimi, Izumi Oki, Toshiyuki Ojima, Yosikazu Nakamura, Shigenori Oguri, Akira Okayama, Yasuhiro Matsumura, Hiroshi Yanagawa

**Affiliations:** 1 Department of Public Health, Jichi Medical School.; 2 Department of Hygiene and Preventive Medicine, Iwate Medical University School of Medicine.; 3 Department of National Nutrition Survey and Health Informatics, National Institute of Health and Nutrition.; 4 Saitama Prefectural University.

**Keywords:** smoking, signs and symptoms, respiratory, correlation, ecologic studies

## Abstract

Background: Although the fact that smoking habits have adverse effects on health, whether the high proportion of smokers elevates the prevalence of symptoms relating to the smoking in a community is still unknown.

Methods: An ecologic study about whole Japan was conducted. Age-adjusted smoking prevalence was calculated using the National Nutrition Survey data from 1986 through 1995 by prefecture and sex. Age-adjusted respiratory symptom prevalence were observed using the National Life-style Survey data in 1995. Correlation among 46 and/or 43 prefectures was examined by sex.

Results: There was a negative correlation between smoking prevalence and wheezing prevalence among males (r=-0.301). Among females, positive correlations were observed on the symptoms of nasal obstruction (r=0.355), nasal discharge (r=0.344), sore throat (r=0.481), cough (r=0.350), sputum (r=0.594), wheezing (r=0.451), palpitation (r=0.363), dyspnea (r=0.587), and frontal chest pain (r=0.472).

Conclusions: Smoking prevalence was deeply related to respiratory symptoms among females in Japan.

Proportion of smokers among Japanese males has been still high and the reduction of smoking prevalence must be overcome. The proportion was 47.4% among males and 11.5% among females in 2000 according to the National Nutrition Survey.^1^ Hazardous effects of smoking to human health are well known.^2^^-^^4^ Even in indirect smoking exposure, the effects are obvious.^5^^-^^7^ We have already observed the unique distribution of smoking prevalence by prefecture, and the observation revealed that smoking prevalence related with standardized mortality rates of some causes of deaths.^8^^,^^9^ In these studies, significant positive correlation with the smoking prevalence was observed for pancreas cancer, decrepitude, unexpected injuries, and traffic injuries in males, and tuberculosis, lung cancer, breast cancer, total heart disease, ischemic heart disease, acute myocardial infarction, pneumonia, chronic obstructive pulmonary disease, asthma, liver disease, and renal failure in females. Thus, prefectures with high proportion of smokers had high standard mortality ratios for some causes of deaths related to smoking. This means excess deaths occurred by smoking. However, health hazardous effects are not only on mortality, but also on morbidity. It is reasonable that incidence rate and prevalence of such diseases as respiratory diseases, cardiovascular diseases, and cancer are high in places where the smoking is prevalent.^10^^-^^12^ Such observations, however, have not been conducted in Japan yet.

A health promotion plan named “Healthy Japan 21” was kicked off by the national government in 2000. Local governments are now starting their own health promotion plans based on the Healthy Japan 21, and they should be developed in a community. On this point of view, data about relationship between health-hazardous exposures and disease-outcomes not only in an individual but also in a community are required. Without such data, making community-based health plans is difficult. The planners should know how much health-hazardous events, e.g., lung cancer deaths, decrease in a certain change of the exposure, e.g., decrease of smoking prevalence, in the community. Because of the abovementioned situation in Japan, ecologic studies as well as analytic epidemiologic surveys based on an individual are important for health planning.

Although, many analytic epidemiologic studies confirmed that smoking affected human health and symptoms, in the current study, we observed the relationship between the smoking prevalence and proportions of some clinical symptomes on a prefecture basis in Japan.

## METHODS

A total of 98,574 persons responded to a questionnaire about smoking habit in the National Nutrition Surveys for 10-year period between 1986 and 1995. The calculation methods and data have already been published.^8^^,^^9^ Briefly, indirect methods to adjust the age distribution were used, and a standardized morbidity ratio of current smokers was obtained by prefecture and sex. In the National Life-style Survey in 1995, a total of 246,892 household reports containing information of 724,015 personnel was collected about the clinical symptoms. Of the symptoms obtained by a questionnaire, 9 related smoking; nasal obstruction, nasal discharge, sore throat, cough, sputum, wheezing, palpitation, dyspnea, and frontal chest pain. Same as the calculation of smoking prevalence indices, the symptom prevalence indices were calculated by prefecture and sex, with age-adjusting using indirect methods.

A big earthquake, so called Hanshin-Awaji Earthquake, occurred in 1995; the National Life-style Survey, therefore, was conducted only for 46 prefectures excluding Hyogo prefecture, which was the center of the earthquake. Because of this, the data of symptoms were also only for the 46 prefectures. Three prefectures, Hokkaido, Tokyo, and Osaka, might be outliers because the smoking prevalence were high among females. Thus, we observed correlation coefficients excluding these 3 prefectures for females (n=43) as well as the whole observations (n=46).

For the both surveys, we used individual data obtained from the Statistics and Information Department, Minister’s Secretariat, Ministry of Health and Welfare of the Japanese government with official permission.

## RESULTS

Prevalence of symptoms were as follows; nasal obstruction is 3.0% (males) and 3.0% (females); nasal discharge is 3.2% (males) and 3.4% (females); sore throat is 1.7% (males) and 2.5% (females); cough is 3.9% (males) and 4.2% (females); sputum is 2.9% (males) and 2.3% (females); wheezing is 1.2% (males) and 1.1% (females); palpitation is 1.4% (males) and 2.2% (females); dyspnea is 1.3% (males) and 1.5% (females); and frontal chest pain is 0.8% (males) and 0.9% (females).

**Figure 1.**
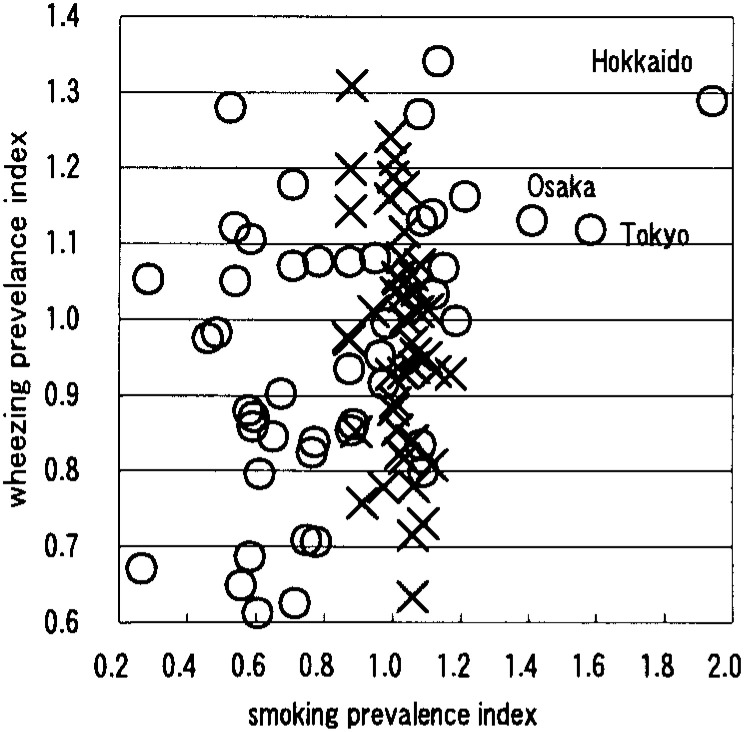
Correlation between smoking prevalence and wheezing prevelance ×: male, r=-0.301 ○: female, r=0.451 (n=46)

**Figure 2.**
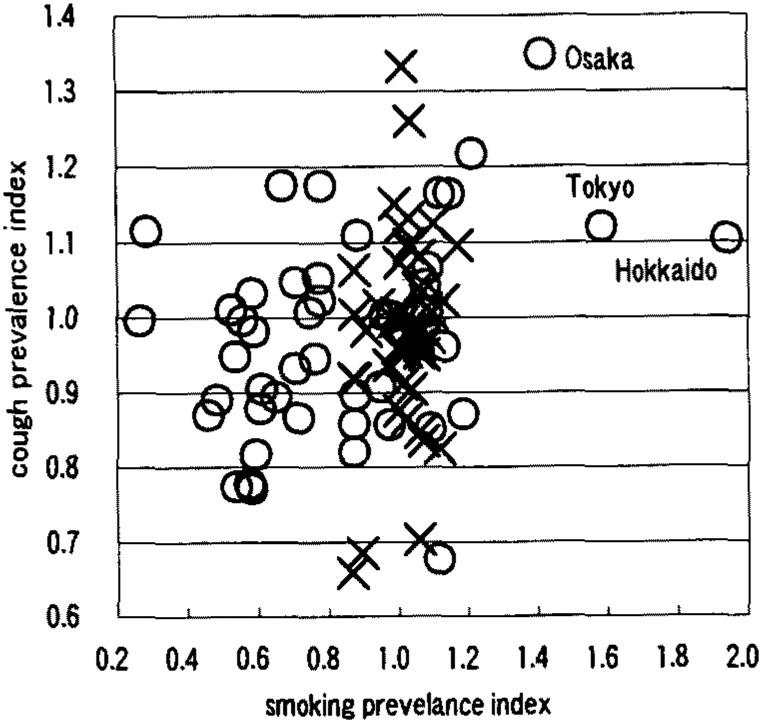
Correlation between smoking prevelance and cough prevalence ×: male, r=0.216 ○: female, r=0.350 (n=46)

**Figure 3.**
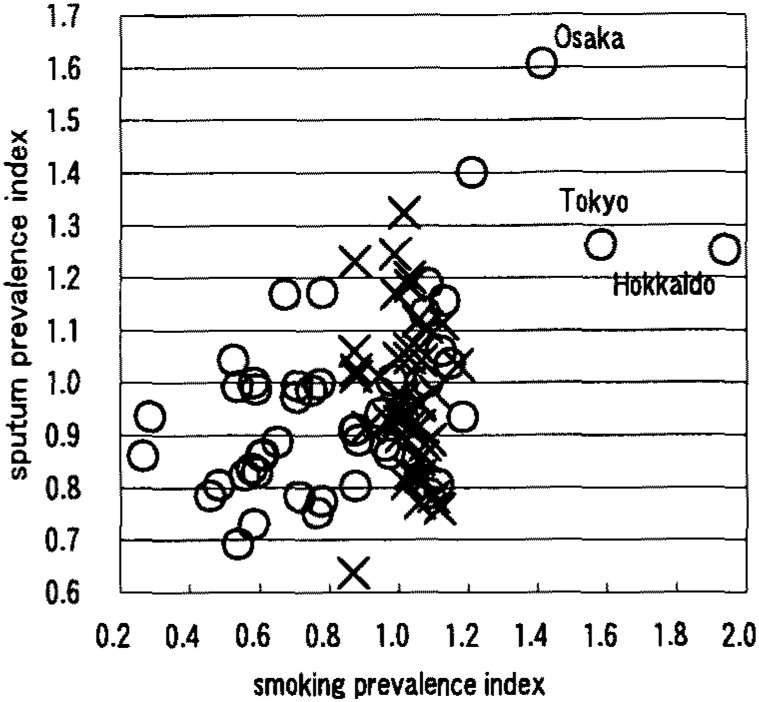
Correlation between smoking prevalence and sputum prevalence ×: male, r=0.014 ○: female, r=0.594 (n=46)

**Figure 4.**
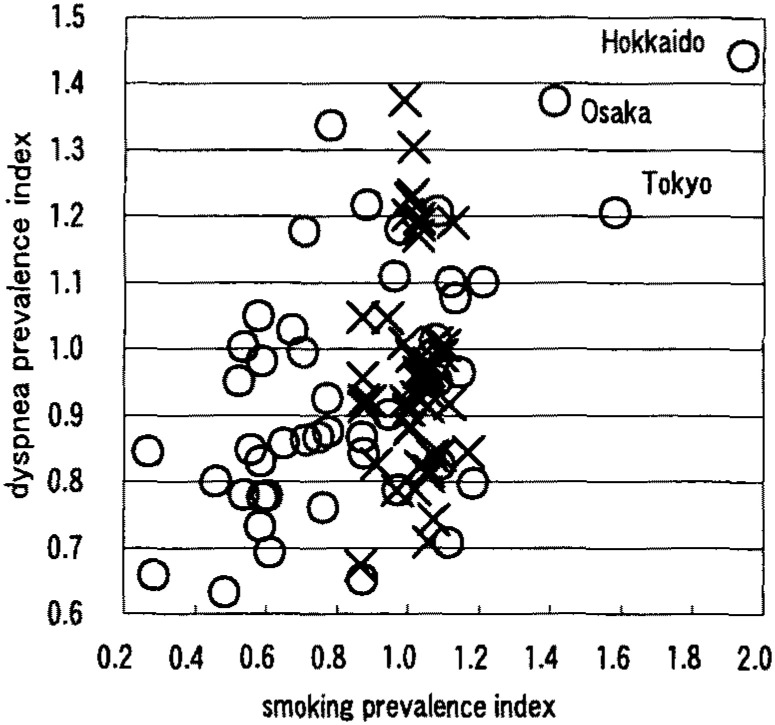
Correlation between smoking prevalence and dyspnea prevalence ×: male, r=0.018 ○: female, r=0.587 (n=46)

Table 1 shows that males had a negative correlation between smoking prevalence and wheezing prevalence (Figure 1). Females had positive correlations on the symptoms of nasal obstruction, nasal discharge, sore throat, cough (Figure 2), sputum (Figure 3), wheezing (Figure 1), palpitation, dyspnea (Figure 4), and frontal chest pain on the observation of 46 prefectures. In the further view of 43 prefectures among females, there were positive correlations on the symptom of sore throat, sputum, wheezing, dyspnea, and frontal chest pain.

**Table 1.** Correlations with smoking prevalence and symptom prevalence: The National Nutrition Survey in Japan (from 1986 through 1995) and the National Life-style Survey in Japan (1995).

Symptom	male (n=46)	female (n=46)	female (n=43)^a^
nasal obstruction	0.052	0.355 *	0.214
nasal discharge	-0.031	0.344 *	0.164
sore throat	0.025	0.481 **	0.298 *
cough	0.216	0.350 *	0.160
sputum	0.014	0.594 **	0.414 **
wheezing	-0.301 *	0.451 **	0.334 *
palpitation	0.073	0.363 *	0.159
dyspnea	0.018	0.587 **	0.362 *
frontal chest pain	-0.089	0.472 **	0.319 *

* : p < 0.05 ** : p < 0.01

a: without Tokyo, Osaka, and Hokkaido

## DISCUSSION

Political strategies against smoking behaviors become important over the world.^15^^,^^16^ Smoking habit apparently has been damaging human health directly and indirectly. We, Japanese, have had clear strategy against the smoking since April 2000. The Ministry of Health and Welfare set up the strategy as Healthy Japan 21,^17^ which was containing many suggestions and achievements to make progression of human health of the Japanese. On the other hand, smoking habits are shifting to younger persons, especially in females.^1^ Proportion of smokers was high in twenties, as were 60.8% of males and 20.9% of females, respectively. It is important in Japan that they begin smoking in younger days before they would be informed the damages to their health by smoking essentially.^18^^-^^20^ In the current study, we can show the association between smoking habit and respiratory symptom by using two large surveys, the National Nutrition Survey in Japan and the National Life-style Survey in Japan. We carried out the reasonable smoking effect to human health by correlation methods so that smoking may cause respiratory clinical symptoms according to smoking prevalence. Among females, all 9 symptoms had positive correlations with smoking indices referring 46 prefectures. Stronger coefficients were observed in 5 symptoms, sore throat, sputum, wheezing, dyspnea, and frontal chest pain. Among males, reasonable positive correlations with smoking prevalence indices were observed on 6 symptoms; nasal obstruction, sore throat, cough, palpitation, and dyspnea. Contrarily, negative correlations among males were nasal discharge, wheezing, and frontal chest pain. If the results were accepted simply, negative correlations between proportions of clinical symptoms and smoking prevalent indices were interpreted as discrepancy of smoking effects.

Smoking indices among females spread between 0.27 and 1.94, and those among males were between 0.87 and 1.17. Widely spreading of the indices among females might introduce the significant correlations. In other words, there is little difference of the proportion of the males smoker so that correlations were weak among males. Then correlation in females is likely much stronger than that in males. Percentage of smokers among females elevated in several developed countries, such as Finland, France, Norway, Poland, Rumania, Spain, Russia, and Japan.^21^ In Japan smoking prevalence among females increased in urban areas, such as Tokyo and Osaka.^12^^,^^13^ Further analyses among females without 3 prefectures, Tokyo, Osaka, and Hokkaido, also revealed the positive correlations with smoking prevalence and proportions of respiratory symptoms (Table 1). Our data suggest the following facts. We take a case of cough among females. In Osaka, Tokyo, and Hokkaido with high proportion of smokers, the prevalence of cough was 10-30% higher than average. However, if the smoking prevalence would be same as the national average, the high prevalence of the symptom might diminish. This information is quite useful to make a health promotion plan with arrival goals.

The correlation coefficients between smoking prevalence and proportions of symptoms including apparent signs, such as cough and sputum were positive, reflecting the health damage *in vivo*. Only one symptom, wheezing among males was strongly negative with smoking prevalence exceptionally. Hypotheses are whether the negative correlation could be occurred by chance or whether chronic obstructive pulmonary disease, such as, asthma and chronic bronchitis, would affect the result of negative correlation. The reason for wheezing prevalence making negative correlation with smoking prevalence will be mentioned in further study.

Cough is the most common sign of respiratory symptom. The symptom of cough in smokers has been owned to the hypersensitivity of their airways.^22^^-^^25^ Healthy females has a more sensitive cough reflex than does males.^26^ Cold air stimulation and air pollutants; ozone, nitrogen dioxide, sulfur dioxide, etc. are causes of cough as well.^27^^,^^28^ Moreover, smoking is followed to destroy their alveolar structures and functions,^29^ resulting in developing emphysema that also will be causative for cough.^19^^,^^30^ Smoker’s emphysema is related to amount and duration of smoking.^31^ Chronic obstructive pulmonary disease is progressive on smoking situation, and if lung function would be the obstacle with remarkable airflow defect caused by smoking, it would be irreversible to wholly normal level unfortunately.^31^^-^^34^ However, if the obstacle would be light degree, lung function would recover to almost normal level for its age by abandoning smoking. Sputum is often recognized among smoking people as well.^35^ In view of these mechanisms of the symptoms, our results are reasonable.

The relationship between respiratory symptoms and smoking habits has been argued since 1930s.^2^ There were also clear concerns with respect to whether smoking habit increase clinical respiratory symptoms. In those studies, a small smoking group was compared to a small non-smoking one. In recent days, however, the progression of computer technology makes it possible to achieve large study and to analyze as a descriptive study conveniently. As is mentioned above, many reports suggested smoking had disappointed affections against human health. In the present study, we can presume that smoking elevated the risks of some symptoms in a community. Risks are available through like this ecologic method. Then this descriptive method as is compared the two large studies will play an important suggestive role to make strategy toward the Healthy Japan 21. Such a correlation study is very informative for local governments to make an own strategy against smoking. The Healthy Japan 21 has been treated just in prefecture unit rather than whole Japan. For the Healthy People 2010 in the USA is obviously performed as national project.^36^

Advantages and disadvantages must be considered in the situation of making reference to the National Nutrition Survey and the National Life-style Survey. Advantages are found in this study on the aspect of ecologic correlation. First, almost all biases can be ignored for the reason that these studies were targeted to gather data of nutrition and life-style of people independently. In the period of survey, no attention tends to be pay for the questionnaire about smoking habit or their own clinical symptoms. Selection biases could be small behind of main theme, which is collecting nutrition information and life-style information. Sore throat, cough, wheezing and other respriratory symptoms are well known and major subjective or objective complaints. The random selection method of the subjects makes the borderline between subjective standpoint and objective standpoint unclear. For another advantage, no additional costs were required, because this data were primarily collected and used for other purposes. Although smoking has had a risk for clinical symptoms, the risk has been still unknown in a community because it is one of the reasons that this kind of ecologic study would be very expensive if the study had preformed for only a several purposes.

Disadvantage, otherwise, are accompanied by stratification. Confounding factors will distort the results in any way, because estimates are based on stratification of prefecture unit and standardized by sex and consequently containing many unknown and unexpected factors. Life styles in rural prefectures are different in place of residence. For example, considering the attitude to smoking or alcohol-drinking in females, both of them indicate high levels in Tokyo and Osaka.^37^ It does not mean that smoking females prefer to drinking alcohol but that the smoking prevalence and alcohol-drinking rate are higher in urban life than in rural life independently. Air pollution would affect to the respiratory symptoms.^27^^,^^28^ They are thought to be more frequent in urban prefectures than in rural prefectures. Prevalence of respiratory diseases, especially asthma and chronic bronchitis, is different urban prefectures from rural prefectures.^38^ Dietary habits or tendencies are westernized according to their industrialization by prefecture.^1^ Existences of confounding factors must be taken into account in preparing present data and remain a problem on how to treat confounding factors and their effects in the future.

We have made it clear that smoking state has concerns to respiratory symptoms especially in females on the descriptive epidemiology. To ensure the relationship between smoking and symptoms in future, it goes without saying that the relation between smoking and disease, other habit will give place to smoking habit about which we have to argue.
